# Lympho-epithelial carcinoma of the larynx – the big masquerader of squamous cell carcinoma – a case report

**DOI:** 10.3332/ecancer.2025.1936

**Published:** 2025-06-27

**Authors:** Samapika Bhaumik, Prarabdh Singh, Narapareddy Venkata Dinesh Reddy, Shreya Jain, Sambit S Nanda, Paramita Paul, Ashutosh Mukherji, Satyajit Pradhan

**Affiliations:** 1 Department of Radiation Oncology, Mahamana Pandit Madan Mohan Malviya Cancer Center and Homi Bhabha Cancer Hospital, Varanasi 221005, India; 2 Department of Pathology, Mahamana Pandit Madan Mohan Malviya Cancer Center and Homi Bhabha Cancer Hospital, Varanasi 221005, India; 3 Mahamana Pandit Madan Mohan Malviya Cancer Center and Homi Bhabha Cancer Hospital, Varanasi 221005, India

**Keywords:** lympho-epithelial carcinoma, lymphoepithelial carcinoma of larynx, non-nasopharyngeal lymphoepithelial carcinoma, LEC, laryngeal LEC

## Abstract

**Background:**

Lympho-epithelial carcinoma (LEC) is most commonly found in the nasopharynx, while LEC of the hypopharynx and larynx is rare, with fewer than 50 cases in the published literature. As the non-nasopharyngeal presentations are rare, the clinical course, diagnosis and treatment for this tumour are sparsely reported. Here, we report a rare case report of Epstein-Barr virus negative laryngeal LEC treated with combined modality therapy in a tertiary care centre. We also review the literature regarding currently acceptable treatment strategies.

**Case presentation:**

We present a case of a 59-year-old male who presented with hoarseness of voice and acute onset of respiratory distress. Post emergency tracheostomy for respiratory distress, evaluation with contrast-enhanced computed tomography head and neck revealed cT3N0M0 supraglottic disease. Biopsy revealed poorly differentiated carcinoma. In view of thyroid cartilage erosion, the patient underwent two cycles of neo-adjuvant chemotherapy followed by total laryngectomy, bilateral neck dissection and primary closure. Postoperative histopathology revealed ypT1N0 LEC, with adequate margins and adequate neck dissection. The patient was then treated with adjuvant chemoradiotherapy. 6 months follow-up positron emission tomography/computed comography shows no locoregional disease.

**Conclusion:**

The treatment for rare cases like non-nasopharyngeal LEC is yet to be standardised. However, as seen in our case report, multimodality management including surgery, chemotherapy and radiation therapy seems to be a feasible approach to managing such rare cases of non-nasopharyngeal LEC.

## Background/introduction

The World Health Organisation defines lympho-epithelial carcinoma (LEC) as poorly differentiated squamous cell carcinoma or histologically undifferentiated carcinoma accompanied by a prominent reactive lymphoplasmacytic infiltrate, morphologically similar to nasopharyngeal carcinoma [1]. Various synonymous terms for this type of carcinoma are – undifferentiated carcinoma, undifferentiated carcinoma with lymphocytic stroma, undifferentiated carcinoma of nasopharyngeal type and lymphoepithelioma-like carcinoma [1]. The first case of LEC was reported in 1921 when Schmincke in Germany, as well as Regaud and Reverchon in France, coined the term [2]. LEC is most commonly found in the nasopharynx, while LEC of the hypopharynx and larynx is rare, with fewer than 50 cases in the published literature [2, 3]. As the non-nasopharyngeal presentations are rare, the clinical course, diagnosis and treatment for this tumour are sparsely reported [4]. Ebstein-Barr virus (EBV) as a cause for LEC has been noticed to be unlikely [2].

The knowledge of non-nasopharyngeal LECs is currently limited to case reports and small case series. Here, we report a rare case report of EBV-negative laryngeal LEC treated with combined modality therapy in a tertiary care centre.

## Case presentation

This is a case of a 59-year-old male with no comorbidities, presenting with chief complaints of progressive hoarseness of voice for 3 months and acute onset of respiratory distress. He has been a bidi smoker, smoking 2 bundles/day for 25 years, reformed since 3–4 months. In view of acute stridor and respiratory distress patient was tracheotomised at a private hospital prior to presentation in our centre. After presentation at our centre contrast contrast-enhanced computed tomography (CECT) scan of the face and neck dated 15/3/2023 revealed a midline hypopharyngeal heterogeneously enhancing mass measuring 3.9 × 3.3 × 6.9 cm along the medial wall of both pyriform sinuses. It extended inferiorly to post post-cricoid region and involved the medial wall of the pyriform and aryepiglottic fold on both sides, apex of pyriform sinus on both sides, para glottic space, false vocal cord, true vocal cord, posterior commissure and post cricoid. CECT further suggested cartilage erosion of thyroid cartilage, arytenoid cartilage, cricoid cartilage and the crico-arytenoid joint. There were no radiologically significant neck nodes. Multiple ill-defined central lobular nodules in both lungs were detected, predominantly in the left lower lobe, which were suggestive of infective etiology.

In view of gross radiological cartilage erosion coupled with cricoarytenoid joint involvement and extension to post cricoid region, the patient was planned for two cycles of neo-adjuvant chemotherapy (NACT) followed by reassessment for surgery. Since the patient’s general condition was frail, he was deemed unfit for Docetaxel, Cisplatin, 5-Fluorouracil regimen and instead started on 3 weekly Paclitaxel + carboplatin chemotherapy. Post two-cycle NACT fibre optic laryngoscopy and repeat CECT scan revealed a partial response to therapy. The patient underwent total laryngectomy + bilateral Neck dissection + Primary Closure + TEP under general anesthesia on 12/04/2023.

**Figure 1.**
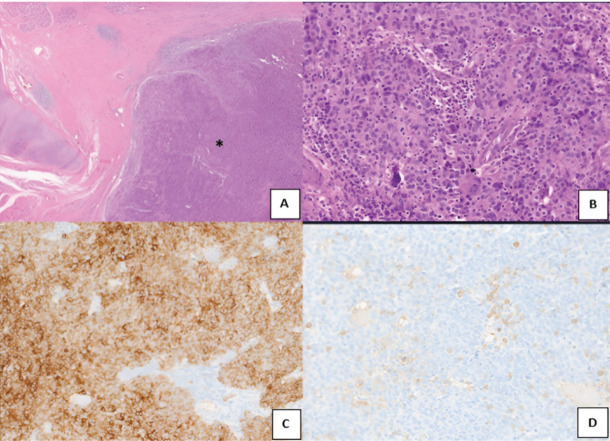
Microscopic images-infiltrating tumour disposed in sheets (*) (a, H&E, ×40), tumour cells surrounded and infiltrated by lymphocytes. (b, H&E, ×200). Tumour cells are diffusely positive for cytokeratin (c, immuoperoxidase, ×100), CD45 highlights the lymphocytes (d, immuoperoxidase,×100).

Postoperative histopathology was suggestive of LEC, at the right supraglottis with a size of 2.5 × 2 × 2 cm. All margins were free of tumour, with the closest margin being the right lateral mucosal margin (1.8 cm from tumour). 49 neck nodes (cervical lymph node bilateral level II a, b, III, IV and central compartment) were dissected and all were negative for metastasis (Figure 1).

**Figure 2.**
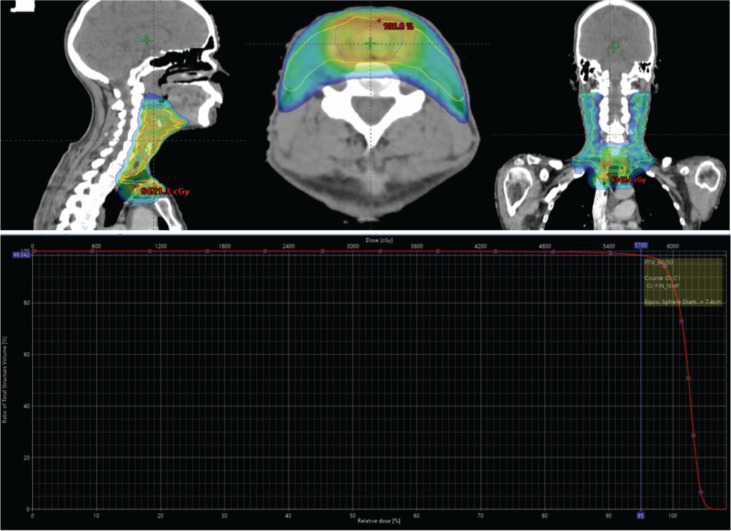
Treatment volumes in sagittal, axial and coronal views; and dose-volume histogram (DVH) of the case during treatment planning.

The patient was planned for adjuvant chemoradiotherapy in view of preoperative NACT. For adjuvant radiotherapy (RT), the patient was simulated in supine position with contrast-enhanced planning computed tomography cuts being taken. The high-risk clinical target volume was contoured including the postoperative tumour bed with expansion based on pre chemotherapy disease extent. In view of prechemotherapy radiological, N0 disease and post operative histopathology showing 0/49 lymph nodes being involved bilateral levels II–IV and prelaryngeal group of lymph nodes were included in low-risk clinical target volume (CTV). Isometric expansion was done on CTVs to achieve respective planning target volumes (PTV). PTV_HR received 60 Gray/30# @2 Gray/fraction, whereas PTV_LR 54 Gray/30#@ 1.8 Gray/fraction using rapid arc technique with intensity modulated RT with simultaneous integrated boost Figure 2. The patient received six cycles of weekly concurrent chemotherapy (cisplatin 35 mg/m^2^) with weekly complete blood count and biochemistry and toxicity monitoring. Acute toxicity post completion of treatment was Grade 1 skin reaction, Grade 2 mucosal and pharyngeal reaction as per RTOG gradings. The patient tolerated the treatment well and has been on follow-up since then. 6 months follow-up positron emission tomography/CT shows no locoregional disease.

## Discussion

The rarity of LEC of larynx and hypopharynx has left the optimal management of these cancers in uncertainty. This warrants an analysis of the pattern of disease and the current standard of management and its correlation with the cancer outcomes, to guide appropriate management of these cancers.

The age of presentation for LEC of the larynx and hypopharynx ranges from 40 to 82 years, with a mean age of 64 years, which differs slightly from the more common Nasopharyngeal LEC (Median ~ 40 years) [2]. Gender distribution is skewed. The disease is ten times more common in males than in females. Caucasian males are more commonly affected as against the Chinese for nasopharyngeal LEC [2].

The etiology of LEC of larynx and hypopharynx is not clear. However, its causal association with EBV, unlike in commoner sites (Nasopharynx, Salivary glands, Oropharynx and Lung), has been noticed to be unlikely. EBV was found to be positive only in 17.2% of LEC cases in larynx and hypopharynx [2]. Human Papilloma Virus association with LEC of the larynx and hypopharynx has been in only 50% of the patients [2]. In contrast, p53 abnormalities may be present in ~ 70% of all patients with LEC of larynx and hypopharynx [2, 5, 6]. Smoking and alcohol were studied as potential risk factors, but a causal association with LEC of larynx and hypopharynx is not established [2].

Diagnosis of LEC poses a challenge; distinguishing it from melanoma or lymphoma becomes difficult without immunohistochemistry markers. Light microscopy shows a background of inflammation with lymphocytes and plasma cells; upon which, large undifferentiated cells are seen. The cell membranes of these undifferentiated cells are mostly ambiguous, giving the appearance of a syncytium (Regaud pattern). Occasionally, individual cells or small cell clusters may be seen (Schmincke pattern). In LEC, immunohistochemical stains for keratin and epithelial membrane antigen are positive, while negative staining for leukocyte common antigen indicating epithelial differentiation [7]. These allow its differentiation from melanoma, which is HMB + and Melan A +; from neuroendocrine tumours, which are Synaptophysin + and Chromogranin +; from smooth muscle tumours, which are desmin + and actin +.

An interesting feature of LEC is the presence of mixed tumour types on histology. It has been found that a substantial proportion of these tumours (25%) can have other histologies admixed with them, squamous cell carcinomas being the most common [2]. This feature is not peculiar to the site under discussion, but has been observed in LEC of the nasopharynx as well. From a reporting standpoint, these tumours are labelled based on the predominant histological pattern, with a note added to indicate the presence of the second histology. However, from a management perspective, there has been no evidence for the need for different management in these two tumour types. Another closely associated entity that has been described in the larynx and hypopharynx is the large cell neuroendocrine carcinoma. The correct identification of this tumour type becomes relevant in that the management of this type differs from LEC, squamous cell carcinoma (SCC). In addition, the prognosis of these tumours is worse, which needs to be communicated to the patient.

Surgery and RT have remained the radical management in these cases. Faisal *et al* [2] had systemically reviewed all reported cases of LEC of the larynx and hypopharynx and found that around 26% of cases were treated with surgery alone. The majority of the patients received surgery followed by adjuvant RT (37%), one-fifth received radiation alone and a small fraction received surgery followed by chemoradiation (15%). The group that underwent surgery alone fared best with a 90% disease-specific survival, mainly owing to the early-stage disease in these patients. Since it has been found that around 25% of these tumours [2, 6] develop distant metastasis, it may be prudent to include chemotherapy in the management plan for these patients. Inclusion of chemotherapy may be as NACT or adjuvant chemotherapy. However, there is no available evidence to guide its practice.

The rate of cervical lymph node metastases in these patients is observed to be around 55%–75%. Hence, a formal neck dissection should be a part of the surgery even in the node-negative neck. If treatment is planned by radical chemoradiation, inclusion of cervical lymph nodes in the treatment volumes becomes mandatory, in line with the management of squamous cell carcinoma of the same region. Although no dedicated data regarding treatment volumes of the primary is available, the current practice of inclusion of the entire larynx in the high dose or intermediate dose region (> 60 Gy @ 2 Gy/#) seems sufficient, in addition to the CTV margins for the tumour.

As per Faisal *et al* [2] review, the 5-year overall survival has been found to be 65% while the 5-year disease-free survival is 68%. Median overall survival was found to be 84 months, while median disease-free survival is 120 months.

## Conclusion

LEC of larynx with cartilage invasion is a rare entity. Evidence pertaining to treatment guidelines, RT volumes and doses are mostly extrapolated from the SCC larynx. Given the fact that our patient tolerated the treatment well and achieved a complete and sustained response, the above approach appears ideal. Multimodality treatment with surgery, RT, chemotherapy with careful consideration to treatment volumes, could yield encouraging results in LEC larynx.

## Patient’s perspective

The patient has been with us on regular follow-up since treatment completion. He has been satisfied that the timely diagnosis of the disease led to prompt treatment onset and interventions in a timely fashion.

## List of abbrveiations

CECT, Contrast enhanced computed tomography; CRT, Chemoradiotherapy; EBV, Epstein-Barr Virus; LEC, Lympho-epithelial carcinoma; NACT, Neo-adjuvant chemotherapy; PTV, Planning target volumes.

## Conflicts of interest

The authors declare that they have no conflicts of interest.

## Funding

No funding for this case report.

## Informed consent for publication

The patient gave written informed consent before the administration of treatment. Written informed consent was obtained from the patient for publication of this case report and accompanying images.

## Author contributions

SB is the principle investigator and main author who, along with PS, NVDR, SJ and SSN, under the guidance of AM and SP, contributed to history and physical examination, data collection, clinical follow-up, literature review, manuscript writing and patient care during treatment and contributed to clinical follow-up and patient care and discussed the results of histopathology with the pathologist, PP. All authors read and approved the final manuscript.
